# Pinocembrin Protects Blood-Brain Barrier Function and Expands the Therapeutic Time Window for Tissue-Type Plasminogen Activator Treatment in a Rat Thromboembolic Stroke Model

**DOI:** 10.1155/2018/8943210

**Published:** 2018-04-22

**Authors:** YinZhong Ma, Li Li, LingLei Kong, ZhiMei Zhu, Wen Zhang, JunKe Song, Junlei Chang, GuanHua Du

**Affiliations:** ^1^Center for Antibody Drug, Institute of Biomedicine and Biotechnology, Shenzhen Institutes of Advanced Technology, Chinese Academy of Sciences, Shenzhen 518055, China; ^2^Beijing Key Laboratory of Drug Target and Screening Research, Institute of Materia Medica, Chinese Academy of Medical Sciences and Peking Union Medical College, Beijing 100050, China; ^3^Institute of Radiation Medicine, Chinese Academy of Medical Sciences, Tianjin 300192, China; ^4^State Key Laboratory of Bioactive Substance and Function of Natural Medicines, Institute of Materia Medica, Chinese Academy of Medical Sciences and Peking Union Medical College, Beijing 100050, China

## Abstract

Tissue-type plasminogen activator (t-PA) remains the only approved therapy for acute ischemic stroke but has a restrictive treatment time window of 4.5 hr. Prolonged ischemia causes blood-brain barrier (BBB) damage and increases the incidence of hemorrhagic transformation (HT) secondary to reperfusion. In this study, we sought to determine the effect of pinocembrin (PCB; a pleiotropic neuroprotective agent) on t-PA administration-induced BBB damage in a novel rat thromboembolic stroke model. By assessing the leakage of Evans blue into the ischemic hemisphere, we demonstrated that PCB pretreatment 5 min before t-PA administration significantly reduced BBB damage following 2 hr, 4 hr, 6 hr, and even 8 hr ischemia. Consistently, PCB pretreatment significantly decreased t-PA infusion-resulting brain edema and infarction volume and improved the behavioral outcomes following 6 hr ischemia. Mechanistically, PCB pretreatment inhibited the activation of MMP-2 and MMP-9 and degradation of tight junction proteins (TJPs) occludin and claudin-5 in the ischemic hemisphere. Moreover, PCB pretreatment significantly reduced phosphorylation of platelet-derived growth factor receptor *α* (PDGFR*α*) as compared with t-PA alone. In an* in vitro* BBB model, PCB decreased transendothelial permeability upon hypoxia/aglycemia through inhibiting PDGF-CC secretion. In conclusion, we demonstrated that PCB pretreatment shortly before t-PA infusion significantly protects BBB function and improves neurological outcomes following prolonged ischemia beyond the regular 4.5 hr t-PA time window. PCB pretreatment may represent a novel means of increasing the safety and the therapeutic time window of t-PA following ischemic stroke.

## 1. Introduction

Thrombolysis with tissue-type plasminogen activator (t-PA) is the only FDA-approved therapy for acute ischemic stroke; however, it has a narrow therapeutic time window of 3 to 4.5 hours after cerebral ischemia onset [1]. Delayed t-PA treatment after prolonged ischemia leads to severe complications such as hemorrhagic transformation (HT), brain edema, and increased mortality [2]. Because of the narrow therapeutic window and potential severe complications, t-PA treatment is applied to less than 5% of ischemic stroke patients [3]. Although the mechanisms underlying t-PA-induced HT are still unclear, blood-brain barrier (BBB) damage can cause HT [2, 4–6]. BBB disruption after ischemic stroke is a dynamic process that is characterized by the initial damage during ischemia and a secondary injury during reperfusion [2]. Prolonged ischemia leads to severe BBB damage that dramatically increases the risk of HT after t-PA thrombolysis. Therefore, developing a novel adjuvant therapeutic strategy to protect BBB integrity and extend therapeutic time window of t-PA during ischemia is critical for improving the outcome of stroke treatment.

Pinocembrin (5,7-dihydroxyflavanone, PCB) is a natural flavonoid compound which is found in honey, propolis, and plants including ginger roots and wild marjoram [7–9]. With the primary target remaining unknown, PCB has shown potent anti-inflammatory and neuroprotective effects through reducing reactive oxygen species (ROS) and apoptosis, modulating mitochondrial function, and protecting the BBB in various animal ischemic stroke models [8–11]. Moreover, PCB ameliorated neuroinflammation and reduced lesion volume in a collagenase-induced intracerebral hemorrhage model and traumatic brain injury model [12, 13]. However, whether PCB is protective in a clinically relevant ischemic stroke model, in which the cerebral artery is occluded by thrombus and reperfused by t-PA thrombolysis, remains unknown. In this study, we observed that PCB was rapidly distributed into the cerebrospinal fluid (5–7 min) and alleviated BBB breakdown induced by t-PA-mediated cerebral ischemia/reperfusion injury in rats. We investigated to what extent and how PCB could mitigate ischemic BBB damage and extend the therapeutic time window of t-PA in a novel rat model of thromboembolic stroke we developed previously [14, 15].

## 2. Materials and Methods

### 2.1. Animal Model of Thromboembolic Stroke

All procedures were approved by the Institutional Animal Care and Use Committee of the Peking Union Medical College and in accordance with the principles outlined in the NIH Guide for the Care and Use of Laboratory Animals. Male Sprague-Dawley (SD) rats (250 to 300 g) were purchased from Vital River Laboratory Animal Technology Co., Beijing. The rats were anesthetized with 3% isoflurane, and the anesthesia was maintained with 1.0% to 1.5% gaseous isoflurane. Rectal temperature was maintained between 37.0°C ± 0.5°C using a feedback-controlled heating system. The MCA of male SD rats was occluded by a thrombus formed within the common carotid artery (CCA) by constant galvanic stimulation, as we previously described [14, 15]. Briefly, the common carotid artery (CCA) was dissected and the galvanic stimulation (1.00 mA) was initiated and sustained for 225 s. The thrombus was smashed 10 times with ophthalmic forceps with a serrated soft tip and flushed into the middle cerebral artery (MCA)/lacunar artery by Willis circulation.

The successful occlusion was confirmed by monitoring cerebral focal perfusion with laser Doppler. Only rats that showed sustained ischemia with less than 25% of the preembolic baselines were included. This embolic stroke model is directly relevant to thromboembolic ischemia in patients, which allowed us to compare the effects of pinocembrin on t-PA's complications under identical and controlled ischemia and reperfusion conditions.

### 2.2. Experimental Design

To investigate the effect of PCB on the progression of BBB damage and its effect on the neurovascular complications of delayed t-PA treatment, we chose four ischemia durations: 2 hours, 4 hours (within the established 3~4.5-hour thrombolytic time window), 6 hours, and 8 hours (outside the window). t-PA was intravenously infused for 30 min and the brain tissue was collected 2 hours after t-PA infusion.

**Figure 1:**
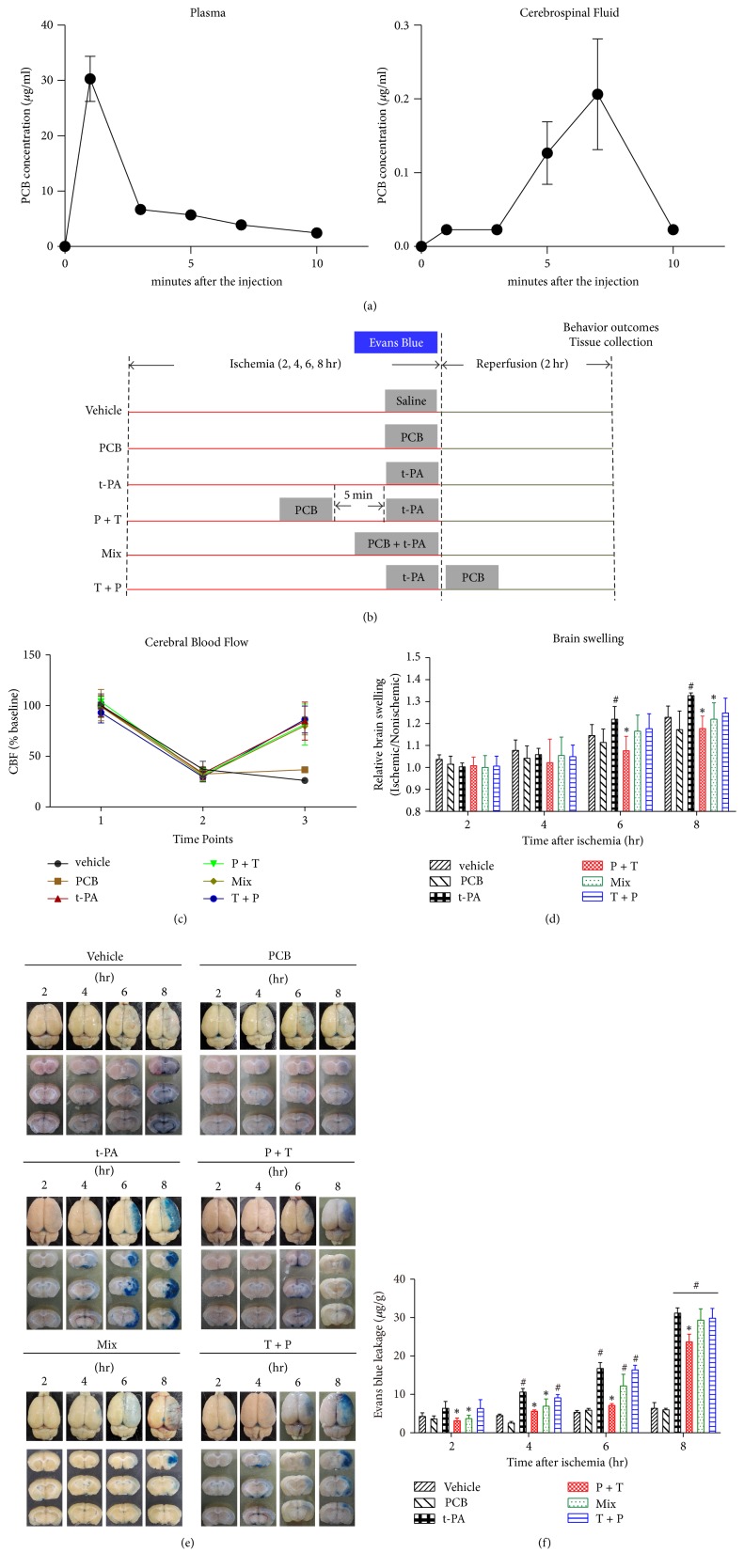
Combination of t-PA and PCB reduces EB leakage in the ischemic brain after 2, 4, 6, or 8 hours of thromboembolic stroke with 2-hour reperfusion. (a) Distribution of PCB in plasma and cerebrospinal fluid (CSF) at 1, 3, 5, 7, and 10 min after the injection. (b) Schematic diagram of the overall experimental design. (c) Focal CBF change during the thrombolysis. Agents were administered at 2, 4, 6, or 8 h after ischemia onset. The time points stand for time (1) before the occlusion, (2) at occlusion, and (3) 2 h after administration. (d) Representative brain slices showing EB leakage in the ischemic tissue. (e) Quantification of EB extravasation is expressed as nanogram per gram of brain tissue (*μ*g/ml). (f) Effect of combination therapy on reducing the brain edema as measured by brain swelling. ^*∗*^*P* < 0.05 versus thromboembolic stroke treated with t-PA; ^#^*P* < 0.05 versus thromboembolic stroke treated with vehicle saline. Data are expressed as the mean ± SEM (*n* = 5).

Rats with successful occlusion were randomly assigned to 6 groups: vehicle, t-PA (*n* = 40), PCB (*n* = 40), P + T (*n* = 40), T + P (*n* = 40), and mixture (*n* = 40). Each group was further divided into 4 subgroups with 2-, 4-, 6- or 8-hour ischemia followed by 2-hour reperfusion. The experimental design is schematically illustrated in Figure 1. Another set of rats were used for long-term (7 days) behavioral tests and mortality: vehicle (*n* = 10), PCB (*n* = 10), t-PA (*n* = 10), and PCB + t-PA (*n* = 10) were administered after 6 hr ischemia. After the initial administration, PCB and P + T group received PCB alone every 24 h, while vehicle or t-PA groups received saline, for another 6 days.

We chose to evaluate the impact of PCB on BBB protection at 2 hours after reperfusion for three reasons: (1) ischemia/reperfusion and t-PA-associated BBB damage occurred rapidly within the first several hours after reperfusion starts [16, 17]; (2) t-PA treatment significantly increased BBB damage as early as the 2 hr poststroke time point [17]; (3) the number of animals needed is minimized as prolonged reperfusion causes high mortality rate.

### 2.3. Reagents Administration

PCB was synthesized by the Department of Medicinal Chemistry of Chinese Academy of Medical Sciences (MW: 255.25; chromatographic purity > 99%). Human recombinant t-PA (alteplase, Boehringer-Ingelheim, Germany) was infused into rats through the right femoral vein at 2, 4, 6, or 8 hours after the occlusion onset, to the P + T, T + P, or mixture (Mix) groups. In the P + T group, PCB was injected 5 min before t-PA infusion. In the T + P group, PCB was injected right after the t-PA infusion, and in the Mix group PCB and t-PA were premixed before the administration. The vehicle used in this study was stroke-physiological saline solution (SPSS). PCB was used as 10 mg/kg* in vivo* and 1 *μ*M* in vitro*. t-PA was used as 1 mg/kg* in vivo* and 10 *μ*g/ml* in vitro*. All the experiments were performed in a blinded manner. The behavior tests and agents' administration were performed by different members of the group to avoid unintentional bias.

### 2.4. Cerebral Distribution of PCB

PCB was injected through the caudal vein in rat. The blood and cerebral spinal fluid were collected at 1, 3, 5, 7, and 10 min after the injection. The analyses were performed on an Agilent Zorbax SB-C18 chromatographic column (5 *μ*m, 4.6 × 250 mm) at 35°C with an Agilent Zorbax SB-C18 precolumn. The chromatographic conditions and sample preparation were in accordance with a previous report [18].

### 2.5. Measurement of Behavior Outcomes

At the end of the 2-hour reperfusion, the neurological deficits test, rotating rod test, forelimb function test, and inclined plane test were assessed 2 hr after the surgery. The neurological deficits test used a modified 5-point Bederson scale to determine the neurological deficits of the rat [19]. The rotating rod test, forelimb function test, and inclined plane test were modified and used to evaluate hemiparesis. The motor coordination of the forelimbs and hind limbs was assessed as previously reported [20–22].

### 2.6. Measurement of Evans Blue Leakage

Evans blue (EB; 4% wt/vol in PBS, 2 mL/kg; Sigma) was administered intravenously into the external jugular vein at the onset of reperfusion. At the end of the 2-hour reperfusion, the rats were transcardially perfused with PBS. The brain was then removed, sectioned, and photographed to visualize EB extravasation. We assessed brain edema by measuring hemispheric enlargement. We also quantitatively assessed BBB disruption by measuring EB contents in ischemic hemispheric tissue, as previously reported [23].

### 2.7. Measurement of Brain Edema

Brain edema was determined by measuring swelling of the ischemic brain tissues. The hemispheric areas of each 2 mm thick brain slice were measured on the digital photographs obtained using ImageJ software (National Institutes of Health), as described previously [24, 25]. Brain swelling was expressed as a ratio of ischemic hemispheric area versus nonischemic hemispheric area.

### 2.8. Quantification of Infarction Volume

TTC staining was performed at 24 hr after ischemia onset. Rats were euthanized and then beheaded after cervical dislocation. The brains were perfused with normal saline, and 8 coronal sections (2 mm thick) were stained with 0.5% 2,3,5-triphenyltetrazolium chloride (TTC) and fixed in 4% paraformaldehyde solution. Infarction volume was measured and analyzed with the Image J software. Edema correction was performed as previously reported [26]. After correcting for edema, the volume of infarction is calculated by summing the infarction area from all slices and multiplying the thickness.

### 2.9. Measurement of MMPs, Occludin, and Claudin-5 in Cerebral Tissue

Brain tissues from the same position as EB measurement were collected after the behavior outcome and edema tests and at 4 hr after ischemia. MMP-2, MMP-9, occludin, and claudin-5 protein levels were measured by Western blot as described [27]. Human MMP-2 and MMP-9 (Chemicon, Temecula, CA, USA) was utilized as the standard (STD) control. All antibodies used in this study were purchased from Cell Signaling Technology, Inc.

### 2.10. Construction and Characterization of BBB Models* In Vitro*

For construction of the BBB models, human cerebral microvascular endothelial cells (cerebEND cells) and astrocyte (CTXTNA2) were cultured to confluence on 24-well collagen-coated Transwell™ tissue culture inserts (0.4 *μ*m pore size, Millipore) in 37°C as was demonstrated in Figure 6(b). Cultures were maintained at 37°C and 5% CO_2_ in a humidified incubator. The growth medium was changed every day, and the cells were grown to a compact monolayer for about 3 days.

To simulate ischemia-like conditions* in vitro*, cocultures were subjected to oxygen-glucose deprivation (OGD) as described [28]. Briefly, cocultures were exposed to OGD for 6 hours, by replacing the culture medium with a glucose- and serum-free medium that had been equilibrated in an anaerobic atmosphere (at <0.1% O_2_, 5% CO_2_, and 95% N_2_) inside a cell incubator. For the reperfusion, cells are transferred to the normal incubator (95% room air and 5% CO_2_) with media change (normal glucose: 5.5 mM) and incubated for another 2 hours. For normoxic controls, cocultures were incubated for 8 hours in glucose-containing, serum-free medium equilibrated with air. BBB permeability was assessed using 10 kDa dextran-conjugated FITC (1 mg/ml) (Sigma-Aldrich) and fluorescence intensity in the lower and upper chamber was measured using a SpectraMax M2e microplate reader (Molecular Devices, Sunnyvale, CA). BBB permeability was calculated as the ratio of lower/upper chamber.

### 2.11. Statistical Analysis

Data were presented as the mean ± SEM. In most cases, statistical analysis was carried out using a one-way ANOVA, with the post hoc Newman–Keuls analysis. For comparison of EB leakage between ischemic hemispheres and nonischemic hemispheres, a paired *t*-test was performed. For comparison of the mortality between t-PA and vehicle or the combination therapy, a chi-square (*χ*^2^) test was performed. A value of *P* < 0.05 was considered to be statistically significant.

## 3. Results

### 3.1. Pharmacodynamic Characteristics of PCB Was in Accordance with Its Distribution in CSF

To make sure PCB reached an adequate brain concentration before t-PA administration, we firstly determined the pharmacokinetic changes of PCB in the plasma and cerebrospinal fluid (CSF). We found that PCB quickly disappeared in the plasma after injection and the peak concentration of PCB in CSF appeared at approximately 7 min after injection (Figure 1(a)). Therefore, we decided to inject PCB 5 min before t-PA administration and reperfusion started (P + T) to maximize the potential protective effects of PCB (Figure 1(b)). PCB was also administered together with or right after t-PA infusion as controls (Mix or T + P, resp.; Figure 1(b)).

### 3.2. PCB Attenuated the Progression of BBB Damage following Prolonged Ischemia

To determine whether PCB could attenuate the progression of ischemic BBB damage caused by delayed thrombolysis, we compared the severity of BBB damage via three different ways of PCB and t-PA coadministration as described above after 2, 4, 6, or 8 hours of ischemia followed by 2-hour reperfusion. Successful occlusion (below 30%) of MCA by thrombus and recanalization (above 70%) by t-PA was confirmed by monitoring the cerebral blood flow via laser Doppler (Figure 1(c)). Figures 1(d)-1(e) shows the EB extravasation after 2, 4, 6, or 8 hours of cerebral ischemia. As expected, EB contents in nonischemic hemispheric tissue were low under all tested stroke conditions. EB content was significantly increased in the ischemic brain tissue and greater EB leakage was seen for longer ischemic duration in both vehicle and t-PA groups. PCB treatment alone did not affect EB extravasation for all 4 ischemic durations. Interestingly, PCB pretreatment 5 min prior to t-PA infusion (P + T) significantly reduced the EB leakage following 2 hr, 4 hr, 6 hr, and even 8 hr ischemia, which was far beyond the 4.5 hr t-PA therapeutic time window, as compared with t-PA infusion alone (Figures 1(d) and 1(e)). Mixed PCB and t-PA administration significantly decreased EB leakage following 2 to 6 hr ischemia as well but was less effective (Figures 1(d) and 1(e)). PCB injection after t-PA infusion appeared to be ineffective.

Brain edema is a major complication of BBB damage. In line with the EB leakage results, t-PA infusion resulted in elevated brain edema as measured by brain swelling after prolonged ischemia (6 hr and 8 hr), which could be significantly inhibited by PCB pretreatment or mixture, but not by PCB posttreatment (Figure 1(f)). These results indicated that t-PA infusion-induced BBB damage and edema occurred rapidly and there was a very short time window for interventional protection of BBB by PCB.

### 3.3. PCB Improved Short-Term Behavioral Outcomes following Prolonged Ischemia

**Figure 2:**
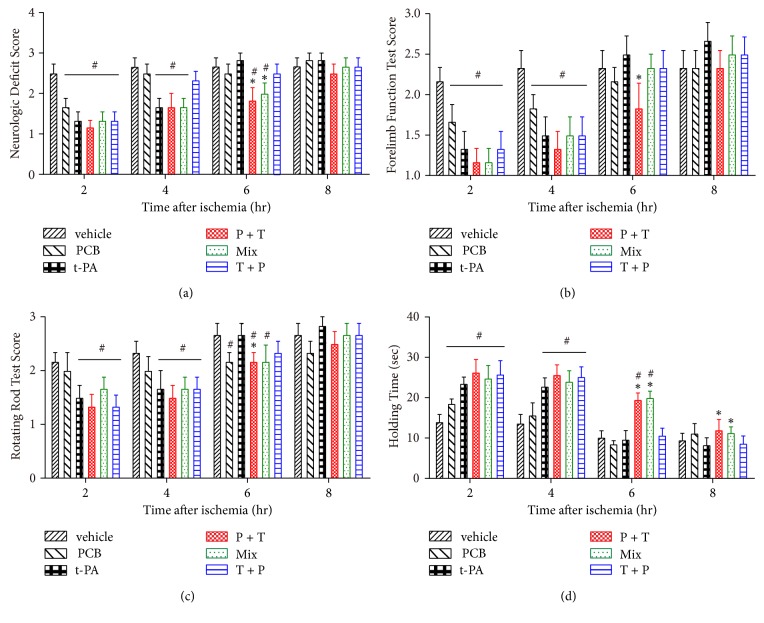
Combination of t-PA and PCB ameliorates behavior outcomes, cerebral edema, and blood supply after 2, 4, 6, or 8 hr of thromboembolic stroke with 2-hour reperfusion. (a)–(d) Neurological deficits test, forelimb function test, rotating rod test, and inclined plane test were examined after 2-hour reperfusion by combination of t-PA and PCB. ^*∗*^*P* < 0.05 versus thromboembolic stroke treated with t-PA; ^#^*P* < 0.05 versus thromboembolic stroke treated with vehicle saline. Data are expressed as the mean ± SEM (*n* = 10).

As to the neurological and behavioral outcomes, we measured the neurologic deficit score, forelimb function, rotating rod test score, and holding time in rats receiving thromboembolic stroke and various treatments. As shown in Figure 2, in rats subjected to 2–8 h of embolism, t-PA infusion alone improved performance at early time points (2 hr and 4 hr) but not at late time points (6 hr and 8 hr) in all four tests at 2 h after thrombolytic therapy (Figure 2). However, PCB pretreatment + t-PA infusion significantly improved performance on all behavior tests at the late time points (6 hr and 8 hr), as compared to t-PA alone. The Mix group displayed a less beneficial effect than P + T group but was better than the T + P group after 6 hours' ischemia. Interestingly, PCB treatment alone also improved the behavioral scores of rats after 2 hr and 4 hr ischemia even if it did not induce reperfusion (data not shown), implying PCB possibly reached the ischemic brain tissue through contralateral circulation.

### 3.4. PCB Pretreatment Reduced Brain Infarction Volume following Prolonged Ischemia

**Figure 3:**
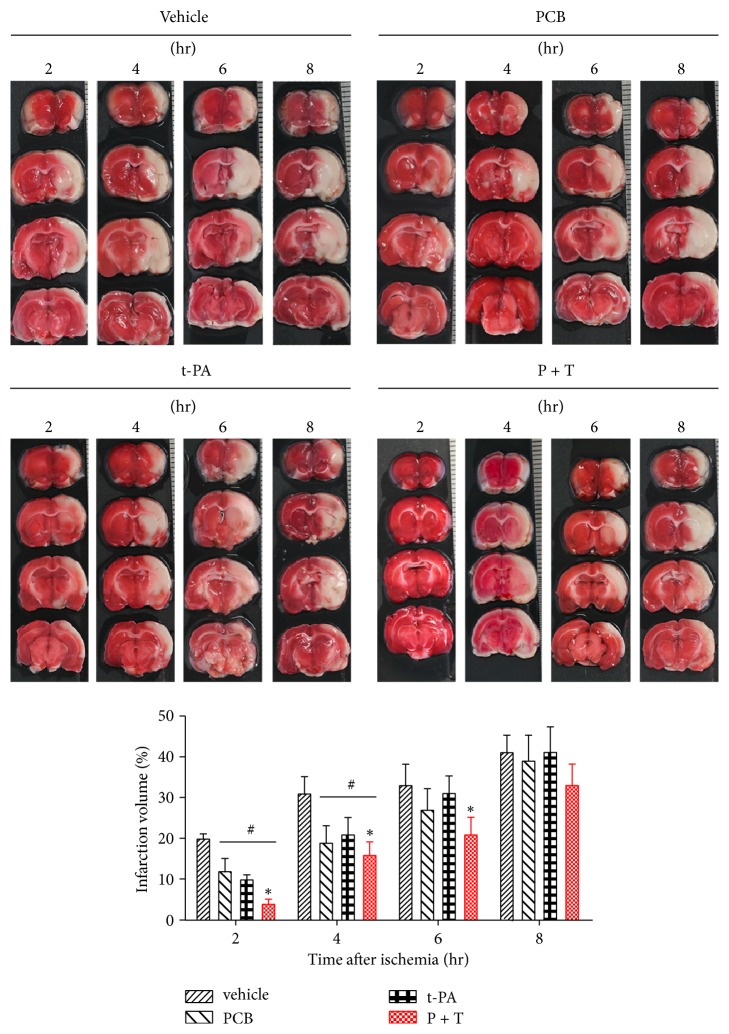
Effect of PCB on the infarction volume. Representative images of TTC-stained brain sections 24 hr after the PCB and/or t-PA therapy. Agents were administered intravenously at 2 hr, 4 hr, 6 hr, or 8 hr after ischemia onset. ^*∗*^*P* < 0.05 versus thromboembolic stroke treated with t-PA; ^#^*P* < 0.05 versus thromboembolic stroke treated with vehicle saline. Data are expressed as the mean ± SEM (*n* = 5).

We next conducted TTC staining to evaluate the effect of PCB on brain infarction. Given that PCB pretreatment (P + T) displayed the most pronounced protective effect, we focused on investigating the therapeutic effect of P + T hereafter. The TTC staining results showed that unlike the BBB damage when t-PA was given within the therapeutic time window (2 hr and 4 hr ischemia), t-PA infusion significantly reduced the infarction volume (Figure 3). When t-PA was given beyond the therapeutic time window (6 hr and 8 hr ischemia), t-PA infusion was not able to reduce brain infarction anymore. However, PCB pretreatment prior to t-PA infusion significantly reduced the infarction volume following 6 hr ischemia (Figure 3, ^*∗*^*P* < 0.01). PCB treatment alone also showed protective effect on infarction after 2 hr and 4 hr ischemia, in line with the behavioral test results (Figure 2).

### 3.5. Repeated PCB Treatment Improved Long-Term Behavioral Outcomes after Delayed t-PA Administration following Prolonged Ischemia

**Figure 4:**
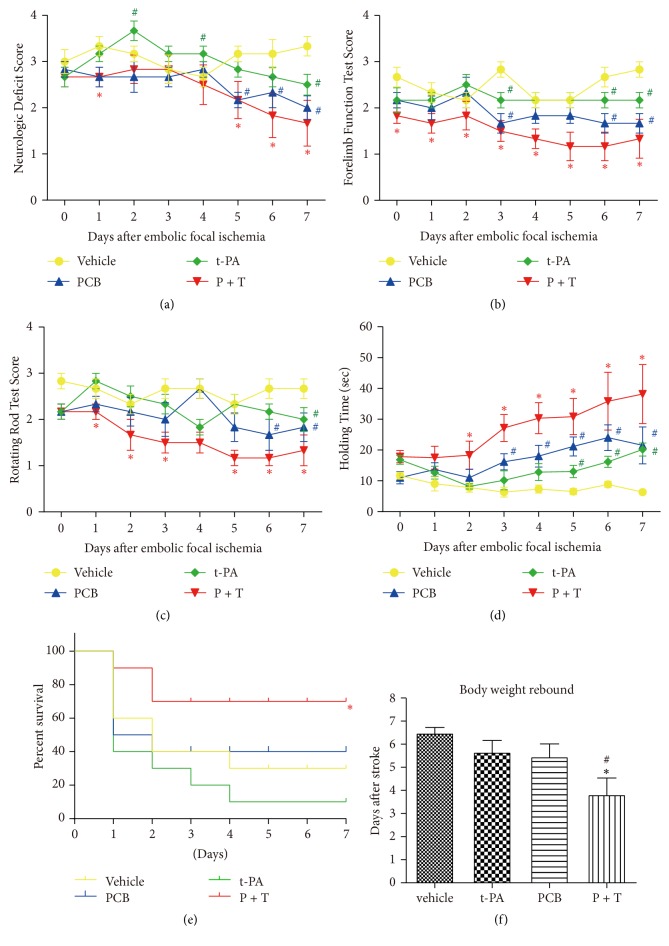
Combination of t-PA and PCB improves long-term behavior outcomes. PCB was administrated every 24 hr for 7 d after the combination therapy at 6 hr after thromboembolic stroke. (a) Neurological deficits test, (b) forelimb function test, (c) rotating rod test, and (d) inclined plane test were examined after the combination therapy at 6 hr after thromboembolic stroke in thromboembolic stroke rats. ^*∗*^*P* < 0.05 versus thromboembolic stroke treated with t-PA; ^#^*P* < 0.05 versus thromboembolic stroke treated with vehicle saline. Data are expressed as the mean ± SEM (*n* = 10). (e) Mortality rates were 70% (7/10) in saline-treated rats and 90% (9/10) in the delayed t-PA treatment group, whereas therapy with PCB + t-PA reduced mortality to 30% (3/10) at 7 d after reperfusion (*n* = 10, ^*∗*^*P* < 0.05 versus t-PA, *χ*^2^ test). (f) Body weights were recorded and the rebound points were compared. ^*∗*^*P* < 0.05 versus thromboembolic stroke treated with t-PA; ^#^*P* < 0.05 versus thromboembolic stroke treated with vehicle saline. Data are all expressed as the mean ± SEM.

The results above clearly showed that one time PCB pretreatment could attenuate delayed t-PA infusion-induced BBB damage and improved neurological functions beyond the standard 4.5 hr time window. We next wondered whether repeated PCB treatment after the initial PCB + t-PA administration could further improve the long-term behavioral outcomes in ischemic rats. PCB + t-PA was administered after 6 hr ischemia as in Figure 1(b); then PCB was injected every 24 hr for 7 days. The results showed that both t-PA infusion alone and repeated PCB treatment alone could modestly improve the long-term behavioral performance (neurologic deficit score, forelimb function, rotating rod test, and holding time; Figures 4(a)–4(d)). Importantly, repeated PCB treatment after the initial P + T coadministration substantially improved the long-term behavioral performance.

In the first two days, a large part of the rats treated with t-PA alone died because of severe hemorrhage and ischemic infarction, consistent with previous reports [29–31]. By contrast, the PCB combination therapy (P + T) animals had a significantly lower mortality as compared with the t-PA group (Figure 4(e)).

As shown in Figure 4(f), rats in the vehicle group displayed a time-dependent body weight loss after thromboembolic stroke with a rebound beginning from day 6. Rats in the t-PA group displayed a similar weight loss, with the lowest weight on days 5 to 6. However, treatment with PCB alone or t-PA + PCB alleviated the weight loss significantly (^*∗*^*P* < 0.05).

### 3.6. PCB Inhibited Tight Junction Protein (TJP) Loss in Ischemic Tissue after Delayed t-PA Administration

**Figure 5:**
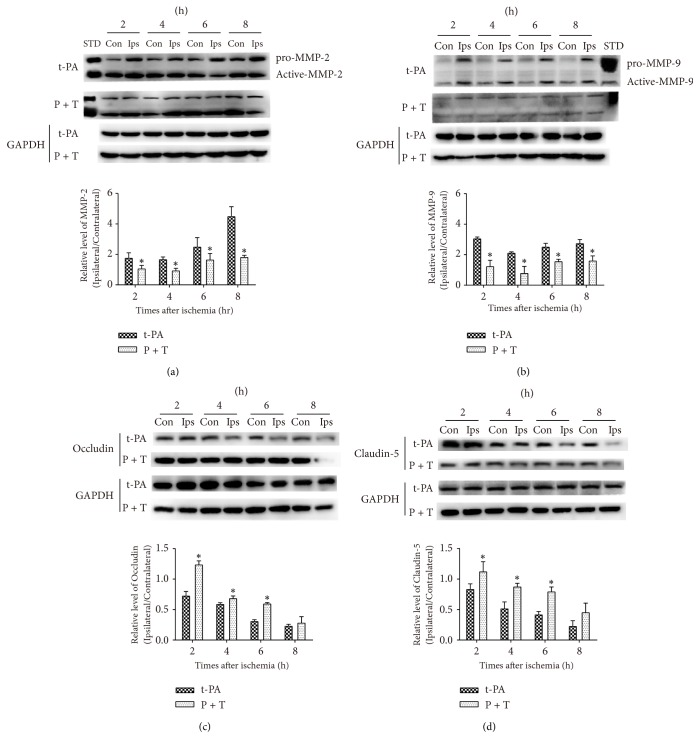
PCB decreased t-PA-induced MMP-2 and MMP-9 activation and TJs (occludin and claudin-5) loss in thromboembolic stroke. ((a) and (b)) the MMP-2 and MMP-9 protein levels in the contralateral (Con) and ipsilateral (Ips) brain tissue in t-PA and PCB + t-PA-treated rats and the quantitative analysis result. STD, the standard of MMP-2 or MMP-9. ((c) and (d)) Western blot analysis of occludin and claudin-5 in cell membrane with different treatments. Rats received t-PA (1 mg/kg) and PCB (10 mg/kg) and tissues were analyzed 4 hr after varied hours of thromboembolic stroke in different groups (*n* = 3; ^*∗*^*P* < 0.05, versus nonischemic, paired *t*-test).

To explore the causes responsible for the beneficial role of PCB, we examined matrix metalloproteinase (MMP) induction and TJP loss under the same experimental regimen as described in Figure 1(b). As shown in Figures 5(a)-5(b), MMP-2 and MMP-9 protein levels were elevated after t-PA administration in ischemic hemisphere as compared to the contralateral hemisphere. Importantly, PCB treatment abolished MMP-2 and MMP-9 induction in the ischemic tissue of t-PA-treated rats following 2 to 8 hr ischemia. Accordingly, ischemia caused a time-dependent loss of occludin and claudin-5 in ischemic tissue after t-PA administration (Figure 5(c)). The PCB pretreatment prior to t-PA infusion reduced the loss of occludin and claudin-5 in rats following 2 to 8 hr ischemia. It is worth noting that PCB only treatment minimally affected the above events (data not shown). The above results demonstrated that PCB might suppress t-PA-mediated BBB damage at least in part by decreasing the induction of MMPs and TJP loss.

### 3.7. PCB Protected BBB Integrity by Inhibiting the PDGF-CC/PDGFR*α* Signaling Pathway

**Figure 6:**
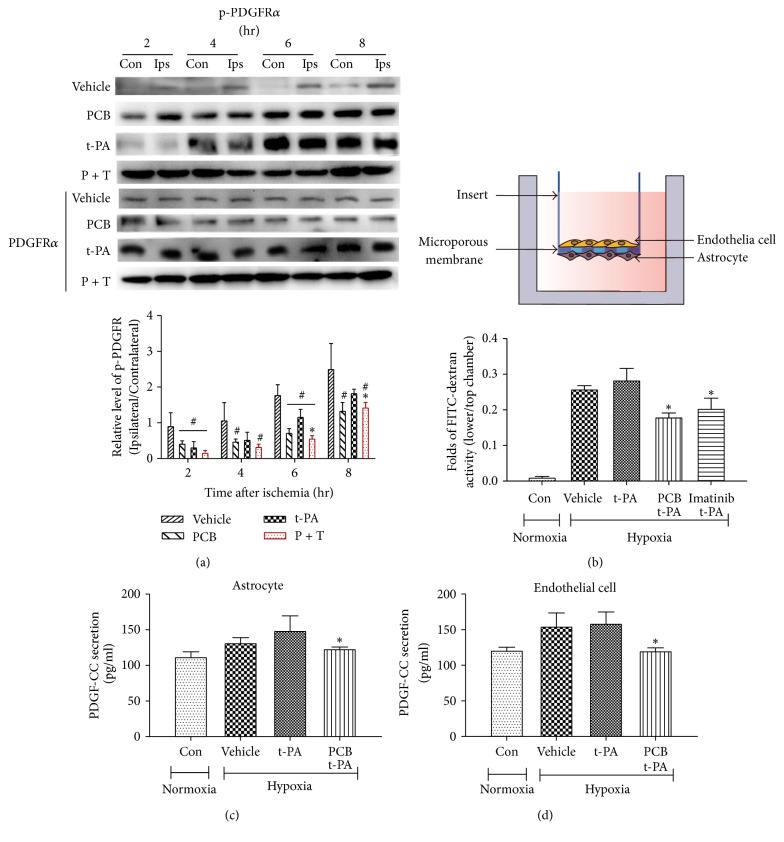
PCB reduced the permeability of BBB model* in vitro* by inhibiting the PDGF-CC/PDGFR*α* signaling pathway. (a) Western blot analysis of phosphorylated PDGFR*α* in cell membrane with different treatments. (b) Schematic of BBB model in vitro by using the Transwell insert. The permeability of BBB model was tested during 6 h of hypoxia/aglycemia and 2 h of reperfusion. (c-d) The contents of PDGF-CC in the culture medium of CTXTNA2 and cerebEND cells (*n* = 3; ^*∗*^*P* < 0.05, versus t-PA; ^#^*P* < 0.05 versus vehicle saline).

Previous studies showed that t-PA induces opening of the BBB through activation of the PDGF-CC/PDGFR*α* pathway [32, 33]. We firstly determined the phosphorylation of PDGFR*α* from the brain tissue of embolic stroke rats. The results showed that the phosphorylation of PDGFR*α* in the P + T treatment group was significantly lower than t-PA group (Figure 6(a)). We next utilized an* in vitro* BBB model and oxygen and glucose deprivation (OGD) to mimic the* in vivo* ischemic stroke condition. We found that after a 6 hr OGD and 2 hr reperfusion the integrity of BBB was compromised, as measured by the fluorescence intensity of FITC-dextran in lower/upper chamber (Figure 6(b)). We found t-PA slightly exacerbated the opening of BBB, whereas PCB or PDGFR*α* inhibitor Imatinib significantly preserved the BBB integrity in the presence of t-PA under OGD situation. Accordingly, we found that secretions of PDGF-CC from astrocytes and endothelial cells were both decreased significantly in the presence of PCB (Figures 6(c)-6(d)), which explained why PCB acted similarly to Imatinib to preserve BBB function. The data above showed that PCB exerts its protective effect on BBB integrity at least in part through inhibition of the PDGF-CC/PDGFR*α* pathway.

## 4. Discussion

In the current study, we comprehensively investigated the protective effects and underlying mechanisms of PCB on t-PA infusion-induced BBB damage in a novel rat thromboembolic stroke model we recently developed. We demonstrated that PCB pretreatment before t-PA administration significantly reduced BBB damage and brain edema and infarction, improved the short-term and long-term behavioral outcomes, and increased survival following 6 hr ischemia, which is beyond the standard 4.5 hr t-PA therapeutic time window. We further showed that PCB preserved BBB integrity by inhibiting degradation of tight junction proteins and activation of the PDGF-CC/PDGFR*α* pathway. Our study presented a potential effective adjunct therapy to increase the safety and the therapeutic time window of t-PA following ischemic stroke.

Tissue plasminogen activator (t-PA) has been demonstrated to be a successful thrombolytic drug in acute ischemic stroke patients but significantly increases the risk of symptomatic HT, which represents the main limitation for thrombolysis [34, 35]. In the National Institute of Neurological Disorders and Stroke (NINDS) t-PA trial [36], the percentage of t-PA-treated patients who developed significant HT following an ischemic stroke was 6.4% compared with 0.6% in the placebo group.

Thrombolysis of the occluded vessel should rescue the affected ischemic zone and improve clinical outcome. However, administering t-PA beyond the 3.5~4.5 hr time window increases BBB injury and then thrombolytic t-PA crosses to the perivascular tissue and causes hemorrhage by interacting with the neurovascular unit [5].

PCB is emerging as a potent neuroprotective molecule that protects neurovascular unit against ischemic stroke injury attributed to its pleiotropic effects on inflammation, ROS, apoptosis, and mitochondrial function [8–13, 37–39]. For instance, PCB protects primary cultured rat cerebral microvascular endothelial cells from the damage induced by oxygen-glucose deprivation/reoxygenation [38]. Other studies indicated that PCB restored BBB integrity or reduced MMP-9 gene expression [37, 40]. Recent studies showed that PCB could significantly mitigate neuroinflammation through inhibiting TLR4 signaling pathway and M1-like microglial polarization in an intracerebral hemorrhage and traumatic brain injury model [12, 13]. With the increasing use of thrombolysis with t-PA, it is important to know if and how PCB affects cerebral hemorrhage associated with t-PA therapy when used in combination with t-PA.

To address the issue, we used three different coadministration approaches of PCB and t-PA in a thromboembolic stroke rat model to elucidate the most curative effect. Interestingly, PCB slowed the progression of ischemia-induced BBB disruption, thus expanding the therapeutic time window of thrombolysis therapy. Compared to t-PA alone, PCB administered 5 min before t-PA infusion (P + T) led to a significant improvement in behavior outcomes and reductions in brain edema and infarction, despite prolonged ischemia durations (6–8 hr).

In this study, we used a novel thromboembolic stroke model that has been a reliable tool in cerebral thrombolysis research, to mimic the clinical ischemic stroke situation. In accordance with other studies, mild signs of bleeding (small petechiae within the damaged area) appeared within 2–4 hr of ischemia in the vehicle group when measured 2 hr after EB injection [41]. Administration of t-PA 2 and 4 hr after occlusion induced recovery of the cerebral blood flow rate up to 70% of initial values. Additionally, a better stroke outcome was observed, as indicated by reduced brain edema and infarction and better behavior test scores. Prolonged ischemia duration, especially in the t-PA administration group, exhibited remarkable differences versus its early counterpart.

BBB breakdown is a common pathological process that occurs after cerebral ischemia-reperfusion and is thought to be a prerequisite for HT and poor treatment outcomes [42]. MMPs are elevated in ischemic brain tissue and critically contribute to BBB disruption, brain edema formation, and cerebral hemorrhage via proteolytic degradation of BBB structural components, including TJPs [6, 43]. In accordance with other studies, our data showed that protein levels of MMP-2 and MMP-9 were induced in an ischemia time-dependent manner, and this change was accompanied by the loss of TJPs occludin and claudin-5 [17]. Administration of P + T ameliorated MMP-2 and MMP-9 induction and TJP loss.

PDGF-CC is a specific substrate of t-PA that binds the PDGFR*α*. During cerebral ischemia, neuronal depolarization can result in a surge of local and exogenous t-PA activity, which in turn leads to continued production of PDGF-CC, persistent activation of PDGFR*α* in the neurovascular unit, and, ultimately, loss of BBB integrity. In our study, we found that P + T treatment inhibited the phosphorylation of PDGFR*α* in ischemic brain tissue compared to t-PA infusion. PCB treatment* in vitro* reduced the secretion of PDGF-CC in astrocytes and endothelial cells under OGD situation, implying a mechanism of how PCB inhibited the PDGF-CC/PDGFR*α* signaling. However, the current limitation is that we still could not locate the direct target of PCB. Future work is warranted to clarify the exact mechanism of how PCB preserves the BBB integrity.

In conclusion, our study provided direct evidence that administration of PCB 5 min before t-PA infusion reduced cerebrovascular permeability and stroke lesion volume as well as neurologic deficits and increased mortality associated with late thrombolysis. PCB could be a potential novel therapy to enhance the safety of t-PA thrombolysis following prolonged ischemic stroke.
